# Depression and hypothyroidism: Literature review and case report

**DOI:** 10.1192/j.eurpsy.2021.884

**Published:** 2021-08-13

**Authors:** P. Coucheiro Limeres, B. De La Torre Cruz, A. Cerame Del Campo, A. Franco Soler

**Affiliations:** 1 Psychiatry, Instituto Psiquiatrico José Germain, Leganés, Spain; 2 Psychology, Hospital Infanta Cristina, Parla, Spain

**Keywords:** hypothyroidism, Depression, mood disorder

## Abstract

**Introduction:**

Multiple neuroendocrine disorders can present themselves through diverse psychiatric symptoms. In the case of hypothyroidism it can manifest itself through mood disorders that will require a comprehensive differential diagnosis.

**Objectives:**

We present a case report and a review of the relevant literature about the relation between mood disorders and hypothyroidism.

**Methods:**

We present the case of a 56-year-old man with no prior psychiatric record who concurring with a grieving process, developed a depressed mood, fatigue, decreased daily activity, and home isolation for months of evolution. He was diagnosed of hypothyroidism and treated with levotiroxine. It was necessary to boost hormonal treatment with antidepressant drugs due to the persistence of the symptoms after the resolution of the hormonal deficit.

**Results:**

The relationship of depression in patients with overt hypothyroidism is widely recognized. Common alterations to both disorders that could make their diagnosis difficult have been observed: existence of psychomotor slowing, attentional and executive disturbance, anxiety, asthenia, weight gain, depressed mood or bradypsychia among others. In the case of subclinical hypothyroidism, certain neuropsychiatric disorders have been linked without having conclusive evidence.

**Conclusions:**

An early screening of thyroid function at the onset of psychiatric symptoms in individuals without prior psychiatric record is essential in the provision of adequate treatment. Clinical improvement has been seen with hormone replacement therapy alone. However, in up to 10% of patients it becomes insufficient, being necessary to complete it with antidepressant drugs for the complete resolution of the condition.

